# Provision of Bilingual Dispensing Labels to Non-Native English Speakers: An Exploratory Study

**DOI:** 10.3390/pharmacy7010032

**Published:** 2019-03-25

**Authors:** Helena Herrera, Murtada Alsaif, Ghalib Khan, Nicola Barnes, Paul Rutter

**Affiliations:** 1 School of Pharmacy and Biomedical Sciences, University of Portsmouth, White Swan Road, Portsmouth PO1 2DT, UK; Nicola.barnes@port.ac.uk (N.B.); Paul.rutter@port.ac.uk (P.R.); 2 Written Medicine, 51 Star St., London W2 1QQ, UK; m.alsaif@writtenmedicine.com (M.A.); mg.khan@writtenmedicine.com (G.K.)

**Keywords:** health literacy, language proficiency, bilingual labels, community pharmacy, medicine related adherence, service implementation

## Abstract

Patients with limited English proficiency living in the U.K. receive prescribed medication labels in English. These patients are at risk of worse health outcomes compared with the general population. This article describes a service evaluation of the use of bilingual dispensing labels to facilitate patient understanding of medicine administration instructions. Recruited patients answered two questionnaires to assess engagement with and understanding of their medicine labels. The first was completed at the point of dispensing, and the second within six weeks. Questionnaires were either self-completed or via facilitation over the telephone. A total of 151 participants completed the first questionnaire, and 130 completed the follow-up. Key findings highlighted the lack of engagement by participants with English-language labels and their reliance on asking for help from pharmacy staff, friends, or family to understand the information. However, when provided with information in their preferred language, they reported high levels of understanding and sought help less frequently from a third party. This study has shown that this service has improved understanding of labelling information in this target group.

## 1. Introduction

Medication non-adherence remains a major obstacle to the effective delivery of healthcare globally [1], and is reported to account for 33% of all preventable drug-related hospital admissions [2]. While reasons that lead to non-adherence with prescribed medication are complex, the inability to communicate has been identified as a key aspect preventing patients from taking their medicines appropriately [3]. Misunderstanding administration instructions and confusing different medicines have been found to be contributing factors [1]. Improving access to adequate administration information can have an impact on medication adherence [4], preventing medication safety incidents, including medicines-related hospital admissions, and reducing associated costs [2,5,6].

These issues are compounded in those patients living in an English-speaking country who are not native English speakers. It is estimated that over one million people in the U.K. [7,8] and 26 million in the U.S. [9] have limited English proficiency. The literature shows that health inequalities and poorer outcomes affect this sector of the population particularly [7,10,11,12].

Despite the significance of these issues, little is known about how communication takes place with patients with limited English [10,13]. Reported work has included the use of patient information leaflets, interpreters and telephone interpreting services [14,15,16,17,18], with the latter two services often provided by unqualified bilingual staff [14,16]. Additionally, there has been work, although limited, on the readability and understandability of bilingual medication labels, with findings suggesting that such labels have an application to practice, through improving participants’ understanding [18,19,20]. Previous literature has highlighted how barriers in understanding information would still be experienced by patients supported by the provision of translated information leaflets [14,15]. Issues with the accessibility of interpreters have also been reported, alongside concerns regarding the reliability of translations when these were not qualified [13,21]. Also, while previous work looking at bilingual medication labels have been undertaken, this has been sparse, looking at simplified, non-standardized medication instructions [18], and labels including pictorial representations [19,20], with no previous studies aiming to explore the perceived impact of standard dispensing labels with bilingual information.

The study reported in this paper was a service evaluation aimed to add to this work by establishing patient acceptability when providing prescribed medication with bilingual dispensing labels to those who do not speak English as their first language.

## 2. Materials and Methods

**Figure 1:**
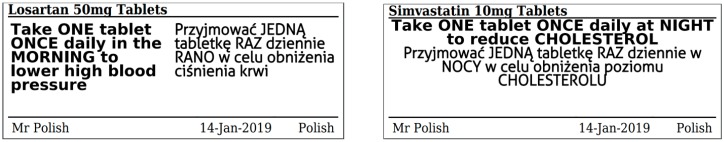
Examples of bilingual dispensing labels.

The service was offered through 12 community pharmacies in London who volunteered to participate after expressions of interest were sought via a local professional pharmacy organization independent from the research team. This organization was approached to invite expressions of interest, as it represented pharmacies in an area that served a large proportion of patients with limited English proficiency. Once the participating pharmacies had been identified, the research team provided training and support to implement the service, which was provided independently under the supervision of registered pharmacists in each of the settings with no affiliation to the research team. The research study was therefore designed by the research team. Dispensing labels were produced that contained both English and the translated language; specifically, the directions of use and any associated warnings (Figure 1). These labels were produced using a cloud-based software similar to the regular dispensing system, which is available throughout the U.K. and in use beyond the settings where the service evaluation took place. The bilingual dispensing labels were not offered in the 12 participating pharmacies prior to them taking part in this work.

**Figure 2:**
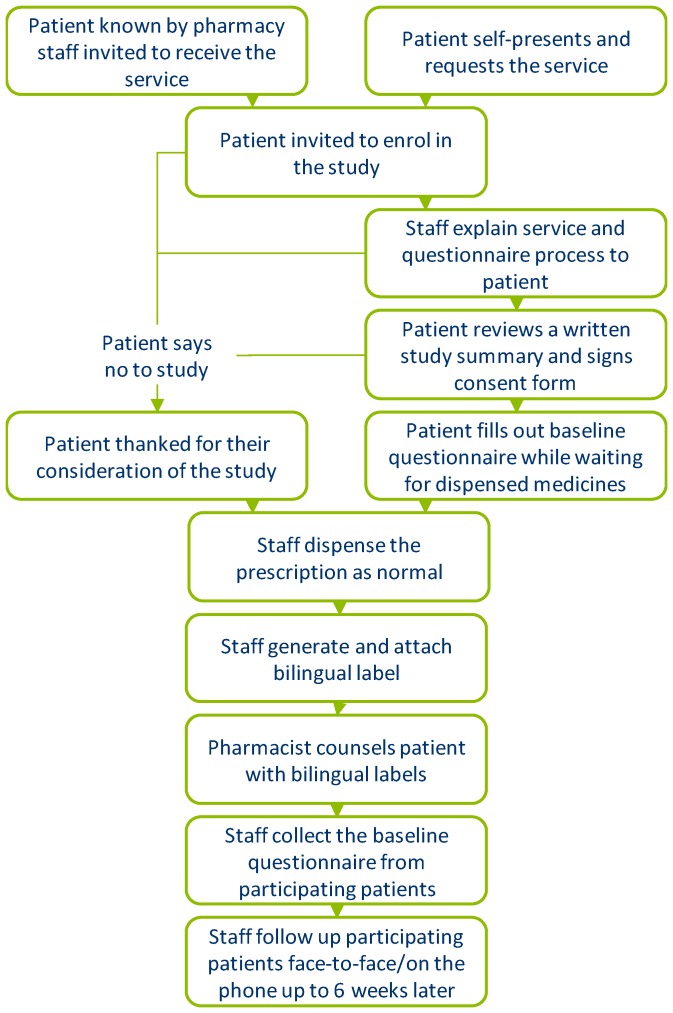
Study protocol and recruitment.

The languages for which the service was available were Arabic, Bengali, Gujarati, Hindi, Polish, Punjabi (Gurmukhi), Somali, and Tamil, and reflected the ethnic groupings that the pharmacies served. Whilst no specific validation of the non-English labels was conducted, the translation software utilized had undergone a four-tier quality assurance process through professional translators, and consisted of: translation; proofreading; quality checking; and testing (by a qualified bilingual U.K. pharmacist). In addition, the U.K. regulator, the General Pharmaceutical Council, was consulted, and confirmed in an oral communication that these labels could be used, and referred the authors to several legal documents pertaining to the labelling of medicines in the U.K., which state that English must be used, but an additional language can be added provided the same information is presented in the additional languages [22,23,24,25]. Figure 2 highlights the recruitment process.

The service was promoted through window poster displays in the pharmacies and informed participants to ask staff for details of the service. Recruitment was therefore predominantly participant-driven, although patients known to staff whose first language was not English were made aware of the service and recruited opportunistically.

Study exclusion were those patients who requested the service but whose preferred language could not be translated by the software, or those who said they could not read their preferred language. Consent to take part in the study was obtained from participants after provision of written information about the project in the person’s preferred language and giving them the opportunity to ask questions about the work to bilingual pharmacy staff. Consent forms were signed by the individuals who agreed to do so prior to participation. These consent forms were also written in the person’s preferred language.

### Data Collection and Analysis

Data were collected from February 2016 to March 2017 from those participants who provided consent to take part in the study as described in Section 2. Patient data were gathered through the completion of two questionnaires, available in the Supplementary Materials: a baseline questionnaire at the pharmacy when they collected their dispensed medication and before they received the bilingual labels for the first time, and a second follow-up questionnaire four to six weeks after. This timeframe was chosen to allow for use of the medicines while aligning follow up with the potential return of patients for repeated medication a month later. Administration of the questionnaires were performed by a nominated staff member (pharmacist or pharmacy support staff) who spoke the participant’s preferred language.

The first questionnaire collected demographic data and questions about their understanding of English labelling information. Additionally, the pharmacist recorded whether the medication was for an acute or chronic condition and whether the supply was new or ongoing.

The second questionnaire was completed either in the pharmacy on a subsequent return visit by the participant or by contacting them via telephone. Participants were made aware on completion of the first questionnaire that they may be contacted in this way.

All questions on both questionnaires were constructed to be simple closed–type questions to facilitate understanding and completion rates.

For both questionnaires, descriptive statistics were used to summarize the data. An enquiry to NHS National Research Ethics Service (NRES) was responded to by confirming that the study was considered a service evaluation and did not require ethical review by an NHS Research Ethics Committee (NRES Queries Line Ref. 04/31). Research ethics approval from the Science Faculty Research Ethics committee at the University of Portsmouth was granted (reference number SFEC 2015—100) prior to commencing this work.

## 3. Results

### 3.1. Initial Questionnaire Data

A total of 151 participants completed the first questionnaire. Data on those who declined to participate were not recorded. Demographic data of participants are shown in Table 1 and highlight that participants were more often middle-aged women or older, who had resided in the U.K. for at least six years and were originally from the Indian sub-continent or Arabic-speaking nations. Unsurprisingly, given the demographic profile, the majority of participants were regular attendees of the pharmacy, receiving repeat medication for the management of long-term conditions.

Participants were asked to rate their abilities in written and spoken English. Findings for both were broadly similar; just under a quarter (23%, n = 35) thought that their understanding of written and spoken English was very good or good, with a slightly higher percentage stating it was poor or very poor (30%, n = 46 for spoken, and 38%, n = 57 for written). The remaining participants rated their ability as fair.

The questionnaire revealed that participants often did not read the English information on the dispensing labels and when they did, they frequently did not understand what was written on them, which resulted in participants often asking for help from someone to explain the information. In this sample, 77% of patients (n = 117) required support from an interpreter “always”, “most of the time”, or “sometimes”. However, the majority still believed they took the medicines as intended (Table 2). Where participants said they did not take the medicine as specified, uncertainty was the main reason (35%, n = 53).

### 3.2. Follow-Up Questionnaire Data

The second questionnaire was completed by 130 of the original 151 participants. Reasons for non-participation in the follow-up questionnaire or whether this questionnaire was completed in the pharmacy or over the phone were not recorded. Of those 130 participants, 98% (n = 128) read the translated information on the dispensing labels. Almost all of these found this information easy to understand all or most of the time (89%, n = 114). Consequently, only 25% (n = 32) of the participants at the follow-up (n = 128) required someone to help them to understand the information on the label in their preferred language. Those who stated needing help (n = 32) were asked who had helped them with this information, and they indicated that these were most often “pharmacy staff” (n = 19), followed by “friends or family” (n = 14), and “others” (n = 3), with participants being able to select more than one option in this part of the questionnaire. They were also asked a series of questions about the impact the translated dispensing labels had on aspects of their adherence. Details of these responses are included in Table 3.

Findings were overwhelmingly positive, with over 80% believing that the translated information helped them to take the correct dose at the right time. Additionally, of those participants who were receiving repeat medication, more than half identified that they had been taking previous medicines inappropriately.

Most participants (79%, n = 101) wished to receive translated labels on a regular basis in the future, and consequently would influence their choice of pharmacy, with the majority of participants (66%, n = 84) stating that they would always or mostly visit a pharmacy offering such a service.

A complete data summary can be found in the Supplementary Materials.

## 4. Discussion

These exploratory results show that people with limited English proficiency struggle to understand the English information provided on dispensing labels. Indeed, a substantial proportion of participants rarely or never read the labels. Participants acknowledged that they frequently sought explanation from others. In this study, this was provided primarily by a bilingual staff member. While this would address some of the communication barriers they experienced, concerns exist around the lack of trained interpreters available in healthcare settings, and the associated risk of miscommunication [13,21]. After receiving the bilingual dispensing labels, participants reported a marked increase in reading the dispensing labels with an associated reduction in further explanation required by someone. It was also noteworthy to find that 62% of people had identified instances where they were taking their medication inappropriately. These findings support other work that shows this population is at high risk of poor health outcomes and susceptible to adverse medicine effects [7,10,11,12].

In the U.K., the use of standard pharmacy dispensing labels containing information in English allows community pharmacies to meet legal requirements, but when these are received by patients with limited English, they seem unable to meet their fundamental purpose. With a substantial and growing population in the U.K. having English as an additional language [7,8], and similar trends elsewhere, there appears to be a need for similar services.

### Limitations

This study was small-scale and exploratory in nature, and therefore does have a number of limitations that need to be acknowledged. Firstly, the results are limited by the nature of self-reporting for understanding medical instructions. This would be an important element for future studies to address, either with qualitative studies on how patients were utilizing this type of service or how it impacted medication-taking behaviour or with adherence-focused quantitative studies. Secondly, we did not objectively assess participant level of English proficiency at recruitment. This needs to be taken into consideration as approximately a quarter of study participants appeared not to have limited English proficiency. Lastly, although a quality assurance mechanism was in place to determine the appropriateness of translated labels, no formal validation was conducted. Cultural differences and how they may impact understanding and interpreting medicines instructions were not explored as part of this work. In addition, the participation numbers were relatively small, affecting generalizability of the results.

This study has shown that patients value the provision of bilingual labels and highlights that medicines may be being taken inappropriately if only English labels are provided. Pharmacies could consider adopting the practice of providing bilingual labels where need exists. Further research would be needed to look at cultural differences and health beliefs that could affect how medicine information is interpreted and understood within each of the languages.

## Figures and Tables

**Table 1:** Demographic data of participants.

Characteristic	Frequency
Gender	
Male	58 (38%)
Female	92 (61%)
Age group	
18–25	7 (5%)
26–40	40 (27%)
41–60	37 (25%)
Over 60	64 (42%)
Length of time in the U.K.	
Less than 1 years	8 (5%)
1–5 years	22 (15%)
6–10 years	29 (19%)
More than 10 years	89 (59%)
Native language	
Arabic	51 (34%)
Bengali	31 (21%)
Gujarati	14 (9%)
Hindi	14 (9%)
Polish	9 (6%)
Punjabi (Gurmukhi)	18 (12%)
Somali	4 (3%)
Tamil	10 (7%)

Not all patients answered all questioned, hence, percentages may not always sum to 100%.

**Table 2:** Participants’ responses on understanding of English dispensing labels and actions taken.

	“Always” or “Most of the Time”	“Sometimes”	“Rarely” or “Never”
Do you read the information on the labels of your medicines (example shown below)?	36% (n = 55)	37% (n = 56)	26% (n = 40)
How often do you understand this information? *	38% (n = 57)	41% (n = 62)	21% (n = 31)
Does someone else help you to understand this information?	39% (n = 59)	38% (n = 58)	23% (n = 34)
How often would you say that you take the right amount of your medicines (for example, the right number of puffs from the inhaler, the right volume of liquid from a bottle or the right number of tablets) and at the right time, as specified by the label? †	58% (n = 88)	27% (n = 41)	11% (n = 16)

* One patient did not answer; † Four patients were “not sure” and two patients did not answer.

**Table 3:** Participants’ responses with regards to the impact on aspects of self-reported adherence when receiving labels in their preferred language.

	“Yes”	“No”	“Not Sure”
Do you think the translated information helped you to take your medicines at the right time as specified on the label?	82% (n = 105)	8% (n = 10)	10% (n = 13)
Do you think the translated information helped you to use the right amount of medicine, as specified on the medication label (for example, the right number of puffs from the inhaler, the right volume of liquid from a bottle, or the right number of tablets)?	83% (n = 106)	6% (n = 7)	12% (n = 15)
After reading the translated information, did you notice you were taking your medicines in a way other than that specified on the medication label? *	62% (n = 46)	30% (n = 22)	8% (n = 6)

* Subset of patients who only received “repeated” medicines, not “new”, “both”, or “not sure”, n = 74.

## Supplementary Materials

The following are available online at https://www.mdpi.com/2226-4787/7/1/32/s1, Two study questionnaires and complete data summary which shows responses to all questions in both questionnaires.
