# Supplementary material for: Provision of Bilingual Dispensing Labels to Non-Native English Speakers: An Exploratory Study
Source: Pharmacy (Basel). 2019 Mar 25;7(1):32. doi: 10.3390/pharmacy7010032 (PMC6473342; doi:10.3390/pharmacy7010032)
Supplement: Supplementary file 1 [file pharmacy-07-00032-s001.zip › pharmacy-452751-final-supple/Two Questionnaires.pdf]

Supplementary materials: Baseline questionnaire

"An evaluation of the impact of using translated dispensing labels on medication adherence, safety and patient satisfaction"

Participant number: ________________________ **Pharmacy date stamp**

Is medication:

- ○ Acute
- ○ Chronic
- ○ Both

Is medication:

- ○ New
- ○ Repeated
- ○ Both
- ○ Not sure

Was the patient enrolled onto any other pharmacy services? If so, which one(s)?

- ○ Not sure
- ○ No
- ○ Yes: ____

Please answer all the questions in the way that best applies to you

What is the patient's language?

○ Arabic	○ Bengali	○ Gujarati	○ Hindi
○ Polish	○ Punjabi (Gur)	○ Somali	○ Tamil

1. What is your gender:

- ○ Male
- ○ Female

2. How old are you:

- ○ 18–25
- ○ 26–40
- ○ 41–60
- ○ Over 60

3. How long have you lived in the UK?

- ◯ Less than 1 year
- ◯ 1–5 years
- ◯ 6–10 years
- ◯ More than 10 years

4. How good is your understanding of written English?

- ◯ Very good
- ◯ Good
- ◯ Fair
- ◯ Poor
- ◯ Very poor

5. How good is your understanding of spoken English?

- ◯ Very good
- ◯ Good
- ◯ Fair
- ◯ Poor
- ◯ Very poor

6. Do you read the information on the labels of your medicines (example shown below)?

- ◯ Always
- ◯ Most of the time
- ◯ Sometimes
- ◯ Rarely
- ◯ Never

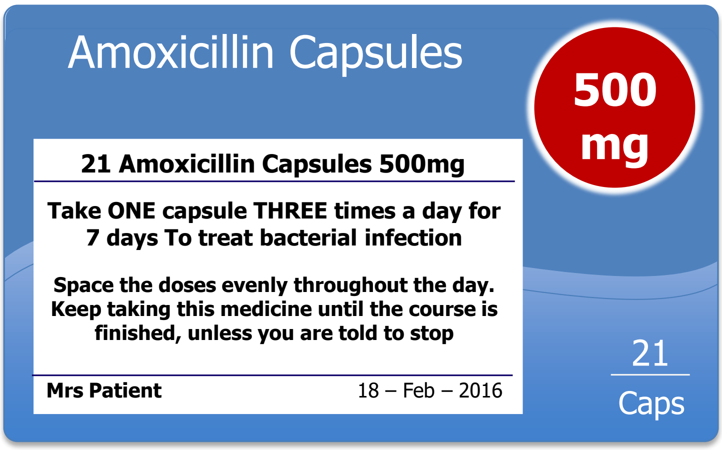

7. How often do you understand this information?

- ◯ Always
- ◯ Most of the time
- ◯ Sometimes
- ◯ Rarely
- ◯ Never

8. Does someone else help you to understand this information?

- ◯ Always
- ◯ Most of the time
- ◯ Sometimes
- ◯ Rarely
- ◯ Never

9. If someone helps you with this information, who would this be? (you can tick more than one answer)

- ◯ The pharmacy staff
- ◯ The surgery staff
- ◯ A friend or relative
- ◯ Others

10. How often would you say that you take the right amount of your medicines (for example, the right number of puffs from the inhaler, the right volume of liquid from a bottle or the right number of tablets) and at the right time, as specified by the label?

- ◯ Always
- ◯ Most of the time
- ◯ Sometimes
- ◯ Rarely
- ◯ Never

11. If there are occasions where you take your medicines in a way other than that specified on the label, what is the reason for this?

- ◯ I choose not to
- ◯ I forget
- ◯ I am not sure how to take them
- ◯ I believe I always follow my doctor's instructions
- ◯ Other reasons

Supplementary materials: Follow up questionnaire

"An evaluation of the impact of using translated dispensing labels on medication adherence, safety and patient satisfaction"

Participant number:

____

Pharmacy date stamp

Please answer all the questions in the way that best applies to you

1. Have you read the translated information on your medicines labels (example shown below)?

○ Yes
○ No

Example:

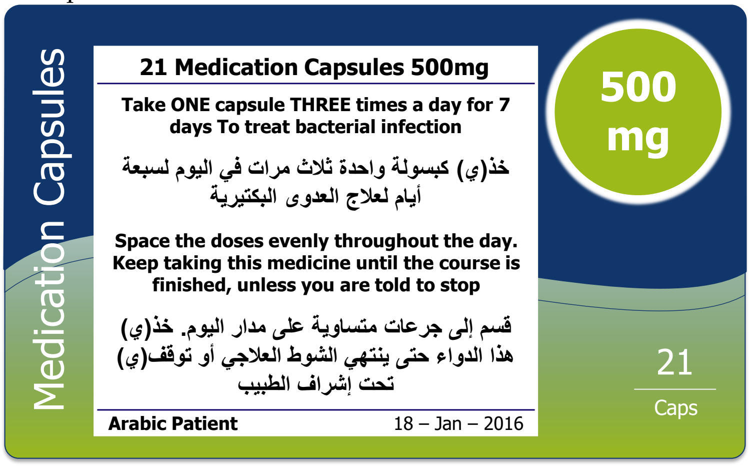

2. Was the translated information easy to understand?

○ Always
○ Most of the time
○ Sometimes
○ Rarely
○ Never

3. Did you need someone else to help you understand this information?

○ Yes
○ No

4. If someone helped you with the Bengali information, please specify who helped you (you can tick more than one answer)?

- ◯ The pharmacy staff
- ◯ The surgery staff
- ◯ A friend or relative
- ◯ Others

5 Do you think the translated information helped you to use the right amount of medicine, as specified on the medication label (for example, the right number of puffs from the inhaler, the right volume of liquid from a bottle or the right number of tablets)?

- ◯ Yes
- ◯ No
- ◯ I am not sure

6 Do you think the translated information helped you to take your medicines at the right time as specified on the label?

- ◯ Yes
- ◯ No
- ◯ I am not sure

7 After reading the translated information, did you notice you were taking your medicines in a way other than that specified on the medication label?

- ◯ Yes
- ◯ No
- ◯ I am not sure

8 Did you visit this pharmacy only because it provides translated labels?

- ◯ Yes
- ◯ No

9 Would you prefer to receive translated labels on a regular basis?

- ◯ Yes
- ◯ No
- ◯ Makes no difference

10 Would the availability of translated labels influence which pharmacy you use?

- ◯ Always
- ◯ Most of the time
- ◯ Sometimes
- ◯ Rarely
- ◯ Never
